# *Prionospio* from the coast of the Iberian Peninsula, with the description of two new species (Annelida, Spionidae)

**DOI:** 10.3897/zookeys.810.26910

**Published:** 2018-12-20

**Authors:** Víctor Hugo elgado-Blas, Óscar íaz-Díaz, José M. iéitez

**Affiliations:** 1 Universidad de Quintana Roo, División de Ciencias e Ingeniería, Chetumal, Quintana Roo, 77010, México Universidad de Quintana Roo Chetumal Mexico; 2 Postgrado en Ciencias Marinas-Instituto Oceanográfico de Venezuela, Universidad de Oriente, Cumaná, Sucre, Venezuela Universidad de OrienteCumaná Cumaná Venezuela; 3 FAUNAMAR LTDA, Consultorías Medio Ambientales e Investigación Marina, Santiago, Chile Consultorías Medio Ambientales e Investigación Marina Santiago Chile; 4 Departamento de Ciencias de la Vida, Universidad de Alcalá, 28871, Alcalá de Henares, Spain Universidad de Alcalá Alcalá de Henares Spain

**Keywords:** key to species, morphology, Polychaeta, spionids, systematics, taxonomy

## Abstract

Five species of *Prionospio* Malmgren, 1867, each with four pairs of branchiae, are studied from coast of the Iberian Peninsula. Two of these species, *Prionospiocristaventralis***sp. n.** and *P.parapari***sp. n.**, are new to science, whereas *P.caspersi* Laubier, 1962, *P.fallax* Söderström, 1920, and *P.ehlersi* Fauvel, 1928 have been previously recorded. *Prionospiocristaventralis***sp. n.** is characterized by having ventral crests present on chaetigers XI–XIX, dorsal crests and low crests on chaetigers X–XXXIV, triangular neuropodial postchaetal lamellae with pointed ventral edges on chaetiger II, oval neuropodial lamellae on chaetiger III, digitiform pinnules on the posterior face of the first and fourth pairs, and branchial pairs II and III are triangular. *Prionospioparapari***sp. n.** is characterized by having rounded neuropodial postchaetal lamellae on chaetiger I, digitiform pinnules on the posterior face of the first and fourth pairs, branchial pairs II and III are cirriform, low dorsal crests on chaetigers VIII–IX, and oval neuropodial lamellae with enlarged dorsal edges on chaetiger III. A key is given to all *Prionospio* species with four pairs of branchiae known from the Iberian Peninsula coastline.

## Introduction

*Prionospio* was established by Malmgren (1867) for *P.steenstrupi* Malmgren, 1867, a spionid species with branchiae on chaetigers II–V, the first and fourth pairs of which are pinnate, and the second and third pairs apinnate. With the discovery of additional species, the diagnosis of the genus was widened to include species with different branchial shapes and arrangements, with the chaetiger upon which the branchiae first arise being particularly relevant. Variability has thus increased, making *Prionospio* a very heterogeneous genus with about 100 species (Sigvaldadóttir 1998). As a result, several authors have suggested that some of these should be reclassified into new genera and/or subgenera (Foster 1971, Blake and Kudenov 1978, Maciolek 1985, Wilson 1990, Sigvaldadóttir 1998).

So far *Prionospiocaspersi* Laubier, 1962, *P.fallax* Södreström, 1920, and *P.ehlersi* Fauvel, 1928 have been recorded from the Iberian Peninsula: the former from Catalonia (Desbruyères et al. 1972) and from off Aveiro (Ravara and Moreira 2013); the second from several localities on the Portuguese coast (Quintino and Gentil 1987; Quintino et al. 1989; Dexter 1992; Pardal et al. 1992; Gil and Sardá 1999, Ravara and Moreira 2013) and the third from the Bay of Biscay (Aguirrezabalaga and Cebeiro 2005), from Coruña (Amoureux 1972, López Jamar and González 1986), from off Aveiro and off Porto (Amoureux 1974, Pardal et al. 1992), and from the South western continental shelf of Portugal (Gil 2011). In this study, we examined material deposited in the Museo Nacional de Ciencias Naturales de Madrid previously identified as *P.caspersi* and *P.fallax*.

**Figure 1.**
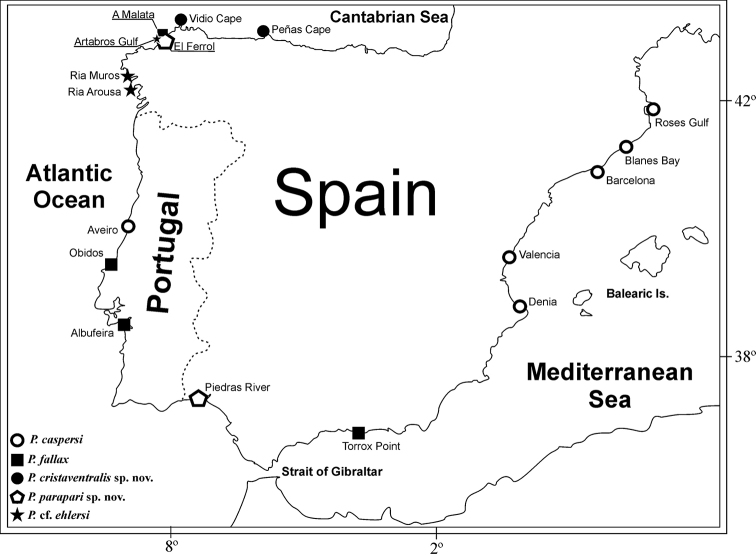
Collection localities of *Prionospio* species from the Iberian Peninsula.

Following the careful comparison between the redescription of *P.fallax* by Sigvaldadóttir and Mackie (1993), redescription of *P.ehlersi* by Mackie and Hartley (1990) as well as the original description of *P.caspersi*, we describe two new species: *P.cristaventralis* sp. n. and *P.parapari* sp. n. An identification key and a map with type localities are provided for all known *Prionospio* species with four branchial pairs from the coastline of the Iberian Peninsula (Fig. 1).

## Materials and methods

The material examined belongs to the collections maintained at the Museo Nacional de Ciencias Naturales de Madrid, Spain (**MNCNM**) and the Alcalá de Henares University, Spain, as well as the personal collection of J Parapar at the University of La Coruña, Spain. Type specimens of the two newly described species are deposited in the MNCNM. For each species, the location where the specimens were collected is indicated in their respective sections.

All material was fixed in 10% formaldehyde in sea water and preserved and stored in 70% ethanol. In order to examine some morphological characters, specimens were dipped first into water and then into methyl green solution for staining. The color fades quickly when specimens are returned to the alcohol solution (Warren et al. 1994). Specimens were measured with a millimeter ruler: body widths refer to the maximum postbranchial body width (including parapodia but not chaetae) at about chaetiger VIII. The eye color mentioned in the descriptions of the species was based on preserved organisms.

## Systematic section

### Spionidae Grube, 1850

#### *Prionospio* Malmgren, 1867 sensu stricto

**Type species.***Prionospiosteenstrupi* Malmgren, 1867, by monotypy.

##### Prionospio caspersi

Taxon classificationAnimaliaSpionidaSpionidae

Laubier, 1962

Prionospio caspersi Laubier, 1962:135–148, figs 1–3.
Prionospio (Prionospio) caspersi : Imajima 1990: 111–114, figs 4–5; Dagli and Çinar 2009: 3.

###### Material examined.

Mediterranean Sea. Valencia: 3 specimens (MNCNM 16.01/4313), 39°43'16"N, 0°10'37"W, coll. G San Martín, April 1998. 1 specimen (MNCNM 16.01/9834), Denia, Alicante, 38°51'17"N, 0°5'37"E, coll. G San Martín, December 1997. 1 specimen (MNCNM 16.01/9836), Denia, Alicante, 38°51'17"N, 0°5'37"E, coll G San Martín, December 1997.

###### Description.

Incomplete specimens, 5.5–8.5 mm long for 30–50 chaetigers, 0.5 mm wide. Prostomium triangular, slightly truncate to convex anteriorly, posteriorly tapered, with narrow caruncle extending to posterior edge of chaetiger I. Two pairs of small black subdermal eyes. Peristomium short, fused dorsally with chaetiger I. Four pairs of branchiae present on chaetigers II–V; pairs 1–3 apinnate, densely ciliated laterally; pair four with numerous digitiform pinnules, on lateral and posterior faces of stems, and naked, smooth distal tips. Notopodial postchaetal lamellae largest in branchial region, lamellae triangular; neuropodial lamellae lanceolate; high dorsal crest across dorsum on chaetiger VII. Sabre chaetae from chaetiger XI, neuropodial hooded hooks from chaetigers XVI–XVII; notopodial hooded hooks from chaetigers XXXII–XXXIII. All hooks with one tooth above main tooth.

###### Remarks.

The specimens examined in this study agree with the original and subsequent descriptions of the species (Laubier 1962, Imajima 1990, Dagli and Çinar 2009). However, there is a slight difference between our specimens and Dagli and Çinar’s specimens: the sabre chaetae were reported as present from chaetiger X by Dagli and Çinar (2009) whereas they first appear on chaetiger XI in our specimens. There are a few further differences between the specimens examined here and the description given by Imajima (1990). Imajima (1990) described the prostomium as being broadly flared anteriorly, with a slight medial indentation and unilimbate chaetae, whereas the specimens in this study have a narrower prostomium that is slightly truncate to convex anteriorly, with bilimbate chaetae. Due, to these morphological differences, the identity of the specimens recorded as *P.caspersi* from Japan by Imajima (1990) should be verified.

###### Habitat.

*Zosteramarina*, sand, muddy sand, depth 3–68 m.

###### Distribution.

Mediterranean Sea: Italy, Venetian Lagoon (type locality); Southern coast of Turkey; Black Sea; Iberian coasts: Aveiro (Portugal), Catalonia, Valencia, Denia (Alicante); Pacific Ocean: Japan.

##### Prionospio cf. ehlersi

Taxon classificationAnimaliaSpionidaSpionidae

Fauvel, 1928

Figure 2A–R

###### Material examined.

Atlantic Ocean. Galicia: 4 specimens (MNCNM 16.01/18424), Ría de Arousa, La Coruña, GA EBS 250, VERTIDOS 04, 42°36'23"N, 8°53'20"W, 26 September 2004, coll. J Parapar; 6 specimens (MNCNM 16.01/18425), GA EBS 200, VERTIDOS 04, 42°36'22"N, 8°52'20"W, 26 September 2004, coll. J Parapar; 17 specimens (MNCNM 16.01/18426), shelf and upper slope off the Artabro Gulf: GA AT 110–4, 43°29'15"N, 8°28'41"W, 25 September 2004, coll. J Parapar; 1 specimen (MNCNM 16.01/18427), DIVA-Artabria 2003: EBS 250, 43°40'14"N, 8°44'3"W, 12 September 2003, coll. J Parapar; 3 specimens (MNCNM 16.01/18428), EBS 1 200E, 43°43'40"N, 8°36'49"W, 12 September 2003, coll. J Parapar; 2 specimens (MNCNM 16.01/18429), EBS 200, 43°43'40"N, 8°36'49"W, 12 September 2003, coll. J Parapar; 25 specimens (MNCNM 16.01/18430), EBS 200, 43°43'40"N, 8°36'49"W, 12 September 2003, coll. J Parapar; 23 specimens (MNCNM 16.01/18431), EB5 200, 43°43'40"N, 8°36'49"W; 3 specimens (MNCNM 16.01/18432), EBS 150 W, 43°31'36"N, 8°43'56"W; 14 September 2003, coll. J Parapar.

###### Description.

Incomplete specimens, 3.0–11.0 mm long, with 13–40 chaetigers, 0.6–1.0 mm wide. Posterior fragment 9.0 mm long for 26 chaetigers, 0.8 mm wide. Color in alcohol pale white. Some specimens with oocytes on chaetigers XXX–XXXIV.

**Figure 2.**
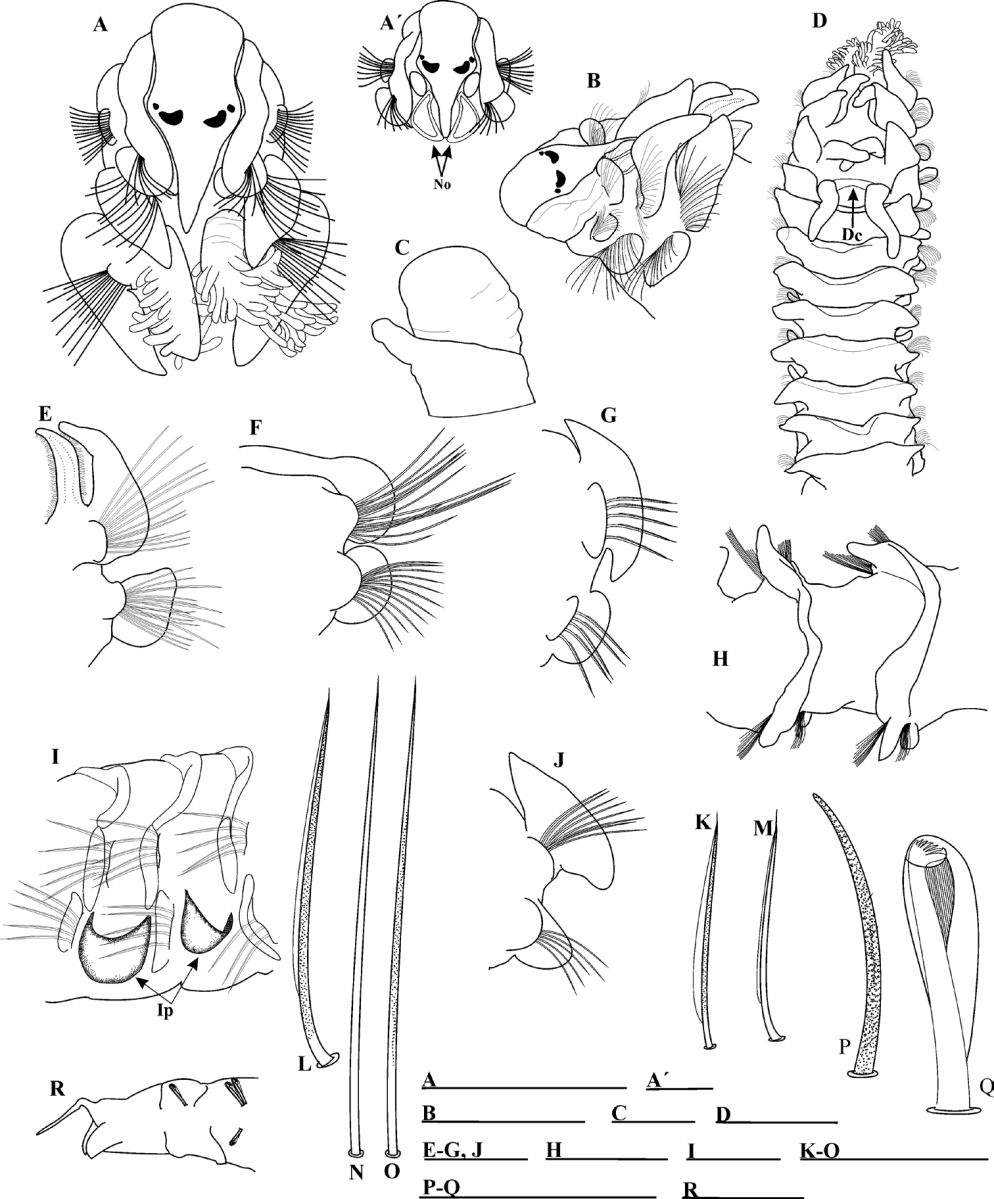
Prionospiocf.ehlersi: **A** Anterior end, dorsal view **A**' Prostomium and detail of nuchal organ **B** anterior end, dorso-lateral view **C** detail of palp showing the short basal sheath **D** anterior end, dorsal view **E** parapodium and branchia of chaetiger 3 **F** parapodium of chaetiger VIII **G** parapodium of posterior chaetiger **H** dorsal crest on middle chaetigers **I** interpodial pouches **J** parapodium of chaetiger V **K** unilimbate notopodial chaeta from anterior row of chaetigers I ,II **L** notopodial capillary chaeta from posterior row of chaetigers I, II **M** smooth, unilimbate, middle notopodial capillary from middle chaetigers **N** long, smooth, alimbate posterior capillary **O** slightly granulated, alimbate capillary from middle chaetigers **P** sabre chaeta **Q** neuropodial hooded hook **R** pygidium, lateral view. Abbreviations: Dorsal cord (Dc), Nuchal organ (No), Interpodial pouches (Ip). Scale bars: 0.9 mm (**A, B, D, H, R**); 0.25 mm (**A**'); 0.07 mm (**C**); 0.5 mm (**E–G, I, J**); 0.04 mm (**K–O, P, Q**).

Prostomium bottle-shaped, rounded anteriorly (Fig. 2A, A', B), posteriorly tapered, with short, narrow caruncle extending to anterior edge of chaetiger II; caruncle with large triangular nuchal organs on either side (Fig. 2A', B). Two pairs of black subdermal eyes, arranged in a trapezoid; anterior pair small, rounded, posterior pair small, crescent-shaped; one paratype without eyes (Fig. 2A, A', B). Palps lost, except in one specimen with palps inserted anterior to nuchal organs: left palp in process of regeneration, with a short basal sheath (Fig. 2C). Peristomium short, collar-like, surrounding prostomium, partially fused dorsally with very large oval notopodial lamellae on chaetiger I (Fig. 2A, B). Neuropodial postchaetal lamellae on chaetiger I small, rounded (Fig. 2A, A', B), smaller than notopodial lamellae. Eversible, sac-like proboscis.

Four pairs of branchiae present on chaetigers II–V, first pair longest and thickest (Fig. 2D); sometimes first and fourth pairs equal in length or fourth pair slightly longer than first. First pair with long, dense digitiform pinnules on lateral and posterior faces, continuing to tip; central stem of branchial pair 1 pinnate, cylindrical, very thick and with a blunt tip (Fig. 2A, D). Pairs 2–4 apinnate; pairs 2 and 3 triangular, thick (Fig. 2D, E), slightly expanded distally, with rounded tips, densely ciliated laterally, shorter than notopodial lamellae and pinnate pair. Pair 4 cirriform (Fig. 2D), basally united by a short, moderate dorsal cord-shape (Fig. 2D); branchiae subequal to, or longer than, notopodial lamellae; some specimens with regenerating branchiae.

Notopodial postchaetal lamellae subtriangular, short on chaetiger II (Fig. 2A, B, D); lamellae on chaetigers III–VII triangular with wide bases (Fig. 2A, B, D); larger and wider on chaetigers III– IV, with long, triangular tips (Fig. 2A, B, E) (in some specimens, the notopodial lamellae on chaetigers III–IV touch each other). Lamellae gradually becoming smaller, rounder and more dorsally directed on chaetiger VIII (Fig. 2F); lamellae on chaetiger XIX subtriangular and becoming angular with ventrally-directed process. Subsequent lamellae progressively decreasing in size, and becoming rounded and wider with ventrally pointed edges (Fig. 2G). Lamellae on posterior chaetigers oval. Notopodial postchaetal lamellae united across dorsum, forming a low dorsal crest, starting on chaetiger VI (Fig. 2D) and continuing as a very low fold or large crest (in large specimens) (Fig. 2H) up to almost end of body. Ventral and dorsal edges of notopodial and neuropodial lamellae touching or overlapping (Fig. 2I) from chaetigers VII–X, up to about chaetiger XX. Anterior notopodial prechaetal lamellae low, rounded; posterior lamellae rudimentary.

Anterior neuropodial postchaetal lamellae large, rounded on chaetiger II (Fig. 2B); very large, more angular and dorsally directed on chaetiger III (Fig. 2E); oval on chaetigers IV–XV (Fig. 2J); subsequent neuropodial lamellae small, rounded up to end of body. Neuropodial prechaetal lamellae low in branchial region (Fig. 2F, J), thereafter increasing in size; some specimens with small ventral rounded lobe like extensions of neuropodial prechaetal lamellae on middle chaetigers, rudimentary on posterior chaetigers. Interparapodial pouches (Fig. 2I) from chaetigers IV–V up to end of fragments, non-reticulated; interparapodial pouches fused with neuropodial prechaetal lamellae.

Notopodial capillaries on chaetigers I–II arranged in two rows, with short, slender, slightly granulated and unilimbate chaetae (Fig. 2K); posterior row longer than anterior one. Notopodial capillaries on chaetigers III–XIII arranged in three rows, anterior row shortest; dorsal chaetae very long and acute (Fig. 2L), ventral chaetae very short and acute; capillaries on middle chaetigers smooth, unilimbate (Fig. 2M); posterior capillaries long, smooth, alimbate (Fig. 2N). Anterior neuropodial chaetigers with granulated, unilimbate capillaries arranged in two rows, anterior row much shorter than the posterior one; capillaries on middle chaetigers slightly granulated, alimbate (Fig. 2O); capillaries on posterior chaetigers smooth, alimbate. Neuropodial sabre chaetae from chaetigers XVII–XX up to two per parapodium, each chaeta long, stout, heavily granulated, alimbate (Fig. 2P). Neuropodial hooded hooks (Fig. 2Q) from chaetigers XVIII–XX, up to 15 per fascicle; notopodial hooded hooks not observed up to chaetiger XL (present in a posterior fragment up to 10 per fascicle); hooks with six pairs of small teeth (Fig. 2Q) above thick, blunt main tooth, and a large principal hood; hooks also appear to possess very striated secondary hoods, producing a feathered effect below main tooth (Fig. 2Q).

Pygidium with one long median cirrus and two short, rounded, lateral lobes (Fig. 2R).

###### Remarks.

Prionospiocf.ehlersi is very similar to the original and subsequent descriptions of *P.ehlersi* (Fauvel 1928, Mackie and Hartley 1990), in that both describe the same prostomial shape, branchial arrangement, and hooded hook structure, and all have interparapodial pouches. However, specimens of this study differ from *P.ehlersi* in that the former have oval (rather than triangular to subtriangular) notopodial lamellae on chaetiger I, the neuropodial lamellae on chaetigers IV–V are oval (rather than rounded), and the second and third branchial pairs are triangular and thick (rather than expanded or swollen distally), a dorsal cord-shape (rather than low crest) on chaetiger V is present, and the sabre chaetae in P.cf.ehlersi are alimbate (rather than sheathed). Mackie and Hartley (1990) reported a variation in the shape of chaetiger I notopodial postchaetal lamellae, and so considered this unimportant. They also noted some parapodial variation around chaetiger XVIII–XX. They did not mention the oval anterior neuropodial postchaetal lamellae, however, Fauvel (1928, fig. 1b) shows a chaetiger IV with neuropodial lamella very similar to that in this study (Fig. 2J). We consider that these differences are important, but we consider it premature to erect a new species with these specimens, without being able to first compare them with Fauvel’s syntype material. The syntypes are deposited at the Museum National d’Histoire Naturelle, Paris under MNHN A438, A449. However, the material cannot be sent abroad for re-examination.

###### Type locality.

Ría de Arousa (La Coruña, Galicia, Spain).

###### Distribution.

Atlantic Ocean. Galicia: Ría de Arousa, La Coruña, shelf and upper slope off the Artabro Gulf, Spain.

##### Prionospio fallax

Taxon classificationAnimaliaSpionidaSpionidae

Söderström, 1920

Prionospio fallax Söderström, 1920: 235–237, figs 135, 144–145; Sigvaldadóttir and Mackie 1993: 207–211, figs 3–5, tables 1–2.

###### Material examined.

Atlantic Ocean, La Coruña: 1 specimen (MNCNM 16.01/15809), A Malata, Ría de Ferrol, 43°29'30"N, 8°14'40"W, coll. J Parapar, 26 October 2000; Mediterranean Sea. Andalucia: 2 specimens (MNCNM 16.01/8756), Punta Torrox, Malaga, 36°43'33"N, 3°57'28"W, coll. G San Martín, February 1995.

###### Description.

Incomplete specimens, 4.5–6.5 mm long for 39–49 chaetigers, 0.5 mm wide. Prostomium bottle-shaped, truncated anteriorly with lateral edges rounded, posteriorly tapered, with a long, blunt caruncle extending to anterior edge of chaetiger II. Two pairs of brown eyes, arranged in a trapezoid; anterior pair small, rounded; posterior pair large, reniform. Four pairs of branchiae present on chaetigers II–V; pairs 1 and 4 equal in size with sparse lateral digitiform pinnules and long naked distal tips; pairs 2 and 3 apinnate, triangular with dense lateral ciliation and sharply pointed tips; shorter than pinnate pairs. Noto- and neuropodial postchaetal lamellae smallest on chaetiger I, rounded in both rami; notopodial lamellae foliaceous, largest on chaetigers III–IV; progressively decreasing in size through chaetigers V–X, becoming rounded. Neuropodial postchaetal lamellae largest in branchial region; lamellae large, subtriangular, ventrally pointed on chaetiger II; those of chaetiger III with dorsally pointed tip; rounded on middle chaetigers, becoming rather inconspicuous on posterior chaetigers. High dorsal crest on chaetiger VII only; no crests on following chaetigers. Interparapodial pouches absent. Sabre chaetae from chaetiger X, up to two per fascicle; neuropodial hooded hooks from chaetigers XII–XIV; notopodial hooded hooks from chaetigers XL–XLIII; hooks multidentate with three to four pairs of small teeth above main tooth and secondary hood.

###### Remarks.

These specimens match the redescription given by Sigvaldadóttir and Mackie (1993), except that we found some specimens with eyes and others without eyes; one specimen had a single large brown eyespot. Possibly, the variation is due to the preservation of the specimens.

###### Habitat.

Silty (mud with much detritus) sediments, depth 25–140 m.

###### Distribution.

Northeast Atlantic, from northern Scotland (Shetland Islands) to the Mediterranean.

##### Prionospio cristaventralis sp. n.

Taxon classificationAnimaliaSpionidaSpionidae

http://zoobank.org/892F5C2D-3F46-4923-94C3-C8FB2DC99A42

Figure 3A–Q

###### Material examined.

Atlantic Ocean. Cantabrian Sea: **Holotype** (MNCNM 16.01/3983), Between Cabo Vidio and Cabo de Peñas, Asturias, Z2 D59, depth 25.6 m, 43°33'30"N, 6°7'1"W, colls. G San Martin and R Acuña Castroviejo, 1998. 1 **paratype** (MNCNM 16.01/3984), depth 34.5 m, 43°33'30"N, 6°7'1"W, colls. G San Martin and R Acuña Castroviejo. 1 **paratype** (MNCNM 16.01/3985), depth 24 m, 43°33'30"N, 6°7'1"W, colls. G San Martin and R Acuña Castroviejo, 1998. 1 **paratype** (MNCNM 16.01/3986), depth 34 m, 43°33'30"N, 6°7'1"W, colls. G San Martin and R Acuña Castroviejo, 1998.

###### Description.

**Figure 3.**
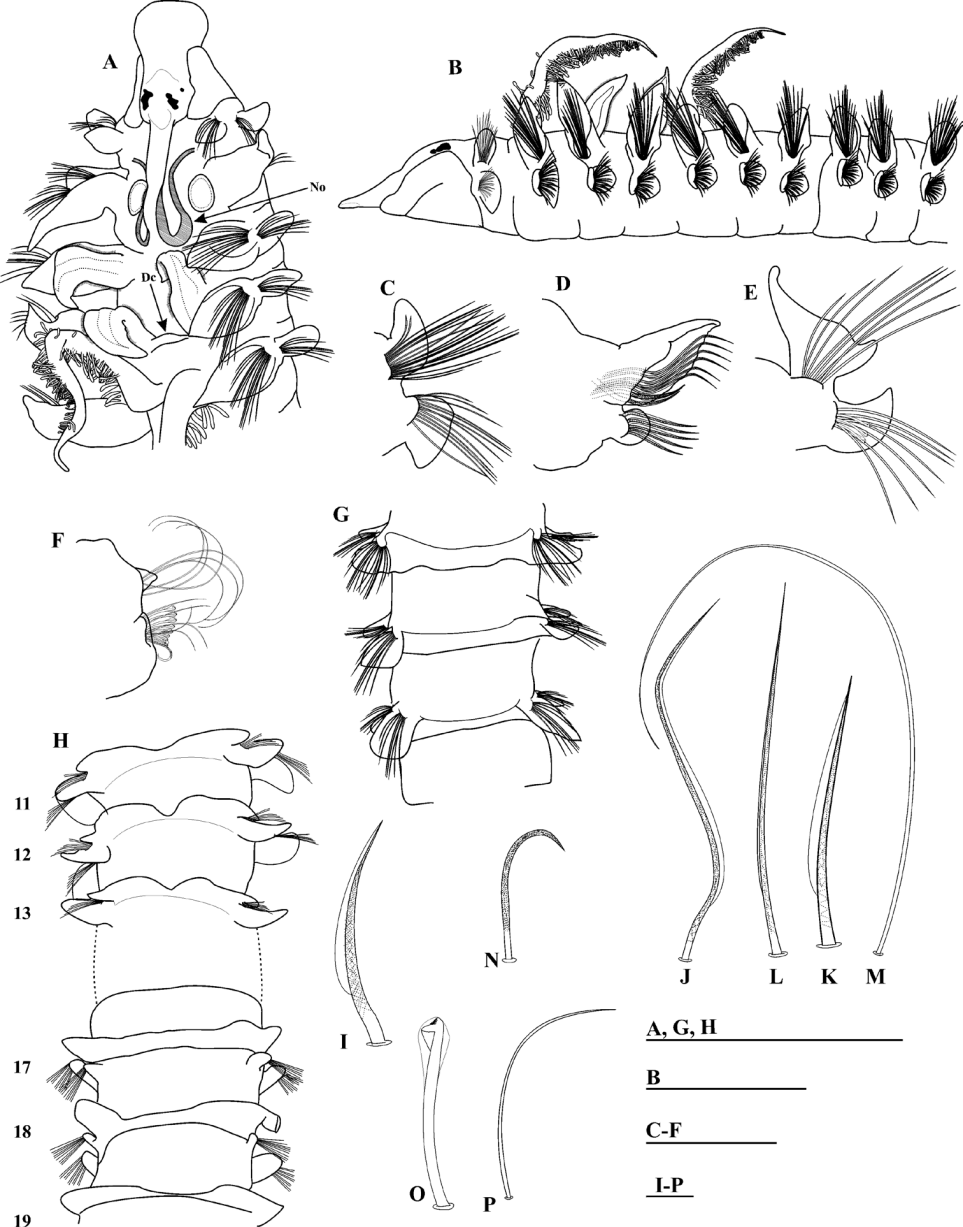
*Prionospiocristaventralis* sp. n. (Holotype MNCNM 16.01/3983: **A, B, G** Paratype MNCNM 16.01/3984: **C, D, E, H, I**): **A** Anterior end, dorso-lateral view **B** anterior end, lateral view **C** Parapodium of chaetiger I (paratype) **D** Parapodium of chaetigers X **E** parapodium of chaetigers 15 **F** parapodium of posterior chaetiger **G** dorsal crest **H** ventral crest **I** short, granulated, unilimbate, notopodial capillary chaeta from anterior row **J** granulated, unilimbate, notopodial capillary chaeta from posterior row of anterior chaetigers **K** short, granulated, unilimbate, notopodial capillary chaeta from anterior row of middle chaetigers **L** granulated, unilimbate, notopodial capillary chaeta from posterior row of middle chaetigers **M** very long, smooth, slender, alimbate, notopodial capillary chaeta from posterior chaetigers **N** sabre chaeta **O** neuropodial hooded hook **P** companion, thin, alimbate capillary chaeta. Abbreviations: Dorsal crest (Dc), Nuchal organ (No). Scale bars: 1 mm (**A, B, G, H**); 0.4 mm (**C–F**); 0.001 mm (**I–P**).

Holotype incomplete, 18 mm long with 34 chaetigers, 1.1 mm wide. Paratypes incomplete, 12.0–13.0 mm long, 22–23 chaetigers, 0.9–1.1 mm wide. Color in alcohol pale white. Prostomium bottle-shaped, broadly rounded, flared anteriorly (Fig. 3A), flattened dorso-ventrally on anterior margin (Fig. 3B), posteriorly tapered, with long, narrow caruncle extending to the posterior edge of chaetiger II, with U-shaped nuchal organs on both sides (Fig. 3A). Two pairs of black subdermal eyes in a trapezoidal arrangement; anterior pair small, rounded, posterior pair very large, crescent-shaped (Fig. 3A, B). Palps lost. Peristomium short, collar-like, surrounding the prostomium, not fused dorsally, with moderate, oval notopodial lamellae on chaetiger I (Fig. 3A–C). Neuropodial postchaetal lamellae on chaetiger I large, oval with ventral edge elongated (Fig. 3A–C), much larger than the notopodial lamellae; notopodial and neuropodial prechaetal lamellae lacking on chaetiger I.

Four pairs of long branchiae present on chaetigers II–V (Fig. 3A, B), first pair longest (Fig. 3B). Pairs 1 and 4 with long, slender, dense digitiform pinnules on posterior face of stems (Fig. 3A, B); branchiae with very long, naked, smooth distal tips, pinnulated basally; pinnule distribution similar on both pairs; pinnules long, slender, blunt in middle and basal regions of branchiae. Pairs 2 and 3 apinnate, triangular, broad, with short pointed tips, densely ciliated laterally (Fig. 3A, B), subequal in length, shorter than pinnate pairs, but slightly longer than notopodial lamellae. Pairs 3 and 4 each united basally by low crest across dorsum (Fig. 3A).

Notopodial postchaetal lamellae on chaetigers II–VII foliaceous with wide bases (Fig. 3A–C), distal halves narrow and elongated; larger and wider on chaetigers III and IV, with long, pointed tips; becoming more oval on chaetigers VIII–X (holotype X) (Fig. 3B, D); lamellae progressively decreasing in size and becoming subtriangular on chaetigers XV– XIX, assuming a more angular form with a ventrally-directed process (Fig. 3E). Subsequent lamellae progressively decreasing in size, and becoming subtriangular (Fig. 3F). Notopodial postchaetal lamellae united across dorsum of chaetigers X and XI forming high dorsal crests (Fig. 3G); on chaetigers XII–XXII/XXXIV (end fragment) forming low dorsal crests. Ventral edges of notopodial lamellae and dorsal edges of neuropodial lamellae not touching on any chaetigers (Fig. 3B–D). Notopodial prechaetal lamellae very large in anterior region, basally fused with notopodial postchaetal lamellae (Fig. 3A, B, D); prechaetal lamellae on chaetigers XI–XV and subsequent lamellae progressively decreasing in size, split, and becoming rounder and smaller (Fig. 3E).

Neuropodial postchaetal lamellae large, triangular, with ventrally-directed process, enlarged on chaetiger II (Fig. 3B); neuropodial lamellae oval on chaetigers III-IX (Fig. 3B), small and rounded on chaetigers X–XIII (Fig. 3D), thereafter, becoming triangular with long, pointed tips (Fig. 3E); subsequent neuropodial lamellae more small (Fig. 3F). Anterior neuropodial prechaetal lamellae short (Fig. 3B, D), progressively increasing in size, becoming rounded and very large on chaetigers XI–XII, connected through well-developed ventral crests forming U-shaped depressions at midline (Fig. 3H); large crests continuing through chaetigers XV–XIX (Fig. 3H); subsequent chaetigers without ventral crests. Interparapodial pouches lacking.

Notopodial capillaries on chaetigers I–V arranged in two rows: anterior row short, heavily granulated, unilimbate (wide limbation), very acute (Fig. 3I); posterior row longer, thinner, more heavily granulated (Fig. 3J); capillaries arranged in three rows on chaetiger VI, similar to anterior chaetae. Notopodial capillaries in middle chaetigers arranged in two rows: anterior row short, granulated, unilimbate (Fig. 3K); posterior row granulated, unilimbate, with very long and pointed tips (Fig. 3L); posterior chaetigers with very long, slender, smooth, alimbate chaetae (Fig. 3M). Neuropodial capillaries on chaetigers I–V arranged in two rows; neuropodial capillaries on chaetigers VI–X arranged in three rows; all capillaries with same structure as notopodial chaetae. Sabre chaetae in neuropodia from chaetiger X, one per fascicle, each chaeta stout, distinctly curved, basally smooth, heavily granulated medially and distally, without sheaths (Fig. 3N). Neuropodial hooded hooks (Fig. 3O) from chaetiger XV, up to 10 per fascicle, accompanied by thin, alimbate capillaries (Fig. 3P). All hooks with six pairs of small teeth above large main tooth, and short, small secondary hoods (Fig. 3O). Notopodial hooded hooks not present on incomplete XXXIV-chaetiger holotype.

Pygidium missing.

###### Remarks.

*Prionospiocristaventralis* sp n. is similar to *P.multicristata* Hutchings & Rainer, 1979, *P.nirripa* Wilson, 1990, *P.nielseni* Hylleberg & Nateewathana, 1991, *P.cornuta* Hylleberg & Nateewathana, 1991, and *P.paradisea* Imajima, 1990 in that all show the same prostomial shape and neuropodial postchaetal lamellae on chaetigers II–III. However, *P.cristaventralis* sp. n. differs from these species due to the presence of ventral crests and dorsal crests limited to chaetigers X–XI, and low dorsal crests on chaetigers XII–XXII/XXXIV (end fragment) compared to low dorsal crests from chaetigers VII to XXV-XXX in *P.multicristata*, low dorsal crests from X–XIII to XXVIII–XXXVII in *P.nirripa*, *P.nielseni* and *P.cornuta* and high crests from X to LX in *P.paradisea*. *Prionospiocristaventralis* sp. n. is also similar to *P.pacifica* Zhou & Li, 2009 in that both species have dorsal and ventral crests or folds. However, *P.cristaventralis* sp. n. can be distinguished from *P.pacifica* by having a prostomium that is rounded anteriorly (instead of being truncate), dorsal crests on chaetigers IV–V (instead of lacking such crests), and ventral crests on chaetigers XI–XIX (instead of only on chaetiger IX). The presence of ventral crests appears to be a unique feature of these two species.

###### Etymology.

The specific name is from the Latin *crista* meaning crests and *ventralis* meaning ventral.

###### Type locality.

Between Cabo Vidio and Cabo de Peñas, Asturias, Spain.

###### Habitat.

Specimens were collected in shallow water (24–34.5 m depth).

###### Distribution.

Atlantic Ocean. Cantabrian Sea: Between Cabo Vidio and Cabo de Peñas, Asturias, Spain.

##### Prionospio parapari sp. n.

Taxon classificationAnimaliaSpionidaSpionidae

http://zoobank.org/EFF7C0D4-5E43-4E9D-9BF6-CB12DB8226BE

Figure 4A–Z

###### Material examined.

Atlantic Ocean. **Holotype** (MNCNM 16.01/18433), Mouth of Piedras River, Huelva: St. D24, 37°12'53"N, 7°7'8"W, coll. L Lopez-Serrano, March 1988. 8 **Paratypes** (anterior fragments) (MNCNM 16.01/18434), St. D22, 37°12'42"N, 7°9'8"W, coll. L Lopez-Serrano, November 1987; 6 **paratypes** (MNCNM 16.01/18435), St. D24, 37°12'53"N, 7°7'8"W, coll. L Lopez-Serrano, March 1988; 2 **paratypes** (MNCNM 16.01/18436), St. D25, 37°12'53"N, 7°7'57"W, coll. L López-Serrano, March 1988; 2 **paratypes** and anterior fragments (MNCNM 16.01/18437), St. D29, 37°6'50"N, 7°4'0"W, coll. L Lopez-Serrano, May 1988; 1 **paratype** and 4 anterior fragments (MNCNM 16.01/18438), St. D38, 37°12'45"N, 7°5'57"W, coll. L Lopez-Serrano, November 1988. Coruña: 1 **paratype** (MNCNM 16.01/12588), Ria de Ferrol, Batel Bay, 1 February 2010, coll. J Parapar; 1 **paratype** (MNCNM 16.01/12569), coll. J Parapar; 64 **paratypes** (MNCNM 16.01/125701); 1 **paratype** (MNCNM 16.01/12572), 43°29'9"N, 08°15'15"W; 1 **paratype** (MNCNM 16.01/12573), 43°29'31"N, 8°10'44"W; 6 **paratypes** (MNCNM 16.01/12574), 43°27'38"N, 08°12'14"W; 5 **paratypes** (MNCNM 16.01/12577), 43°28'51"N, 8°11'13"W; 4 **paratypes** (MNCNM 16.01/12578); 2 **paratypes** (MNCNM 16.01/12579); 1 **paratype** (MNCNM 16.01/12580), 43°29'7"N, 8°10'19"W; 1 **paratype** (MNCNM 16.01/12582), 43°28'46"N, 8°11'45"W; 1 **paratype** (MNCNM 16.01/12583), 43°28'31"N, 8°12'14"W; 1 **paratype** (MNCNM 16.01/12589), 43°28'2"N, 8°16'37"W; 6 **paratypes** (MNCNM 16.01/12575), 43°28'51"N, 8°15'15"W; 1 **paratype** (MNCNM 16.01/15810); 1 paratype (MNCNM 16.01/15802); 1 **paratype** (MNCNM 16.01/15800); 1 **paratype** (MNCNM 16.01/15811), Ria de Ferrol, Laxe Bay, coll. J Parapar; Ria de Ferrol, Redonda Point, coll. J Parapar.

###### Description.

**Figure 4.**
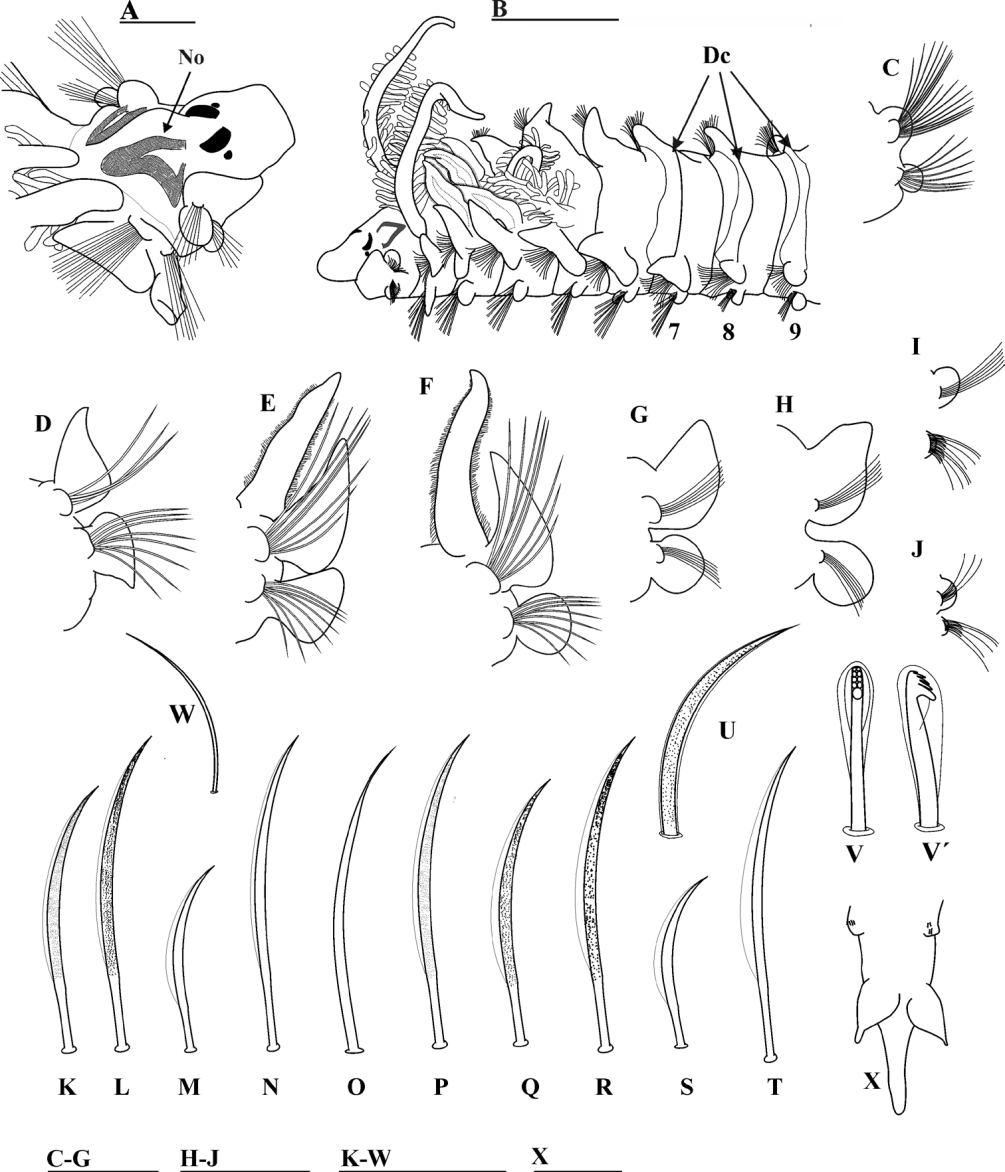
*Prionospioparapari* sp. n. (Holotype MNCNM 16.01/18433: **A, C, L** Paratype MNCNM 16.01/18434: **B**): **A** Anterior end, dorso-lateral view **B** anterior end, dorso-lateral view, showing dorsal crest and crest **C** parapodium of first chaetiger **D–H** parapodium of chaetigers II–VI, frontal view (**D** and **G** with branchiae removed) **I** chaetiger from middle-posterior region **J** chaetiger from posterior region **K** heavily granulated, unilimbate notochaeta from anterior row of anterior chaetigers **L** heavily granulated, unilimbate notochaeta from posterior row of anterior chaetigers **M** unilimbate notochaeta from anterior row of middle chaetigers **N** unilimbate notochaeta from posterior row of middle chaetigers **O** smooth, alimbate chaeta on posterior notopodia **P** slightly granulated, unilimbate neurochaeta from posterior row of anterior chaetiger **Q, R** heavily granulated, unilimbate chaeta from chaetigers II–IX **S, T** smooth, unilimbate neurochaetae from anterior and posterior rows, respectively **U** sabre chaeta **V, V**' neuropodial hooded hooks **W** alternating alimbate capillaries **X** pygidium, ventral view. Abbreviations: Nuchal organ (No), Dorsal crest (Dc). Scale bars: 0.2 mm (**A**); 0.5 mm (**B, X**); 0.4 mm (**G–J**); 0.03 mm (**K–W**).

Holotype complete, 18.0 mm long with 62 chaetigers, 0.4 mm wide. Complete paratypes, 17.0–21.0 mm long with 47–68 chaetigers, 0.2–0.8 mm wide. Incomplete paratypes, 4.0–12.5 mm long with 14–61 chaetigers, 0.2–0.5 mm wide. Color in alcohol pale white. Prostomium bottle-shaped, truncated and narrow anteriorly, widening in mid-region (Fig. 4A), posteriorly tapered, with long, blunt caruncle extending to anterior edge of chaetiger II (Fig. 4A); caruncle with large V-shaped nuchal organs on either side (Fig. 4A, B). Two pairs of brown-black subdermal eyes (holotype brown), arranged in a trapezoid; anterior pair small, posterior pair very large, both pairs crescent-shaped (Fig. 4A, B) (one specimen lacks eyes). Palps lost. Peristomium moderate in size, collar-like, surrounding prostomium, fused dorsally with large, rounded notopodial lamellae on chaetiger I (Fig. 4A, C). Neuropodial postchaetal lamellae on chaetiger I large, rounded (Fig. 4C), less than half the size of notopodial lamellae.

Four pairs of branchiae present on chaetigers II–V (Fig. 4B, C); first pair longer than fourth pair, up to 1 time and a half longer than the fourth pair. Pairs 1 and 4 with long, slender, dense digitiform pinnules arranged along posterior face of the stems, pinnules thick, long, blunt in middle region of branchiae (Fig. 3B); branchiae with very long, naked, smooth, distal tips (Fig. 4B). Pairs 2 and 3 apinnate, cirriform, long, densely ciliated laterally, with pointed tips (Fig. 4E, F); subequal in length, up to 3 times shorter than pinnate pairs, but longer than notopodial lamellae (Fig. 4B, E, F).

Notopodial postchaetal lamellae triangular and slender on chaetigers II–VI (Fig. 4D–H); largest on chaetigers III–IV, with short triangular tips (Fig. 4E, F); becoming wider on chaetigers V–VI (Fig. 4G, H) and thereafter progressively decreasing in size; small, rounded on middle and posterior chaetigers (Fig. 4I, J). Notopodial lamellae united across dorsum, forming a high dorsal crest on chaetiger VII and low dorsal crests on chaetigers VIII–IX (Fig. 4A); some specimens without dorsal crests on chaetiger IX, subsequent chaetigers lacking crests. Ventral and dorsal edges of notopodial and neuropodial lamellae touch only on chaetiger III (Fig. 4E). Notopodial prechaetal lamellae moderate and oval in branchial region (Fig. 4E–G), not basally fused with notopodial postchaetal lamellae, inconspicuous thereafter (Fig. 4H–J).

Anterior neuropodial postchaetal lamellae rounded (Fig. 4C, F–H) throughout, except on chaetigers II–III; lamella on chaetiger II triangular, large, with ventral edge enlarged, pointed (Fig. 4D); lamella on chaetiger III oval with dorsal edge enlarged (Fig. 4E); second and third pairs larger than other neuropodial lamellae; subsequent neuropodial lamellae small and rounded on middle chaetigers (Fig. 4I), and rounded lobes on posterior chaetigers (Fig. 4J). Neuropodial prechaetal lamellae low in anterior region (Fig. 4C–H), rudimentary throughout. Interparapodial pouches lacking. All anterior and middle notopodial chaetae arranged in two rows. Notochaetae on chaetigers I–IX heavily granulated, unilimbate (Fig. 4K, L); posterior row longer, more heavily granulated (Fig. 4L) than anterior row (Fig. K); both rows of chaetae on middle notopodia thin, smooth, unilimbate (Fig. 4M, N); anterior row shorter (up to half) (Fig. 4M) than posterior row (Fig. 4N); posterior notopodia with smooth, alimbate chaetae (Fig. 4O) arranged in one row. All neuropodial capillaries arranged in two rows; capillaries on chaetiger I slightly granulated, unilimbate (Fig. 4P); chaetigers II–IX with heavily granulated, unilimbate neurochaetae (Fig. 4Q, R); posterior row more heavily granulated (Fig. 4R), with limbation wider than for notochaetae. Neurochaetae smooth, unilimbate on subsequent chaetigers, those on anterior row up to three times shorter and wider (Fig. 4S) than those on posterior row; limbation of posterior row wider than that of the anterior one (Fig. 4T); chaetae on posterior neuropodia arranged in one row. Neuropodial sabre chaetae from chaetigers X–XII (holotype X), up to two per fascicle, each chaeta stout, curved, heavily granulated, bilimbate (Fig. 4U). Neuropodial hooded hooks (Fig. 4V, V') from chaetigers XI–XV (holotype XIII), up to eight per fascicle, alternating with long, thin, alimbate capillaries (Fig. 4W). Notopodial hooded hooks on chaetigers XXIX–XXXVIII (holotype XXXIII), up to six per fascicle, alternating with up to two thin, alimbate capillaries, hooks on posterior chaetigers longer than those on middle chaetigers; all hooks with four pairs of small teeth above main tooth, and large secondary hoods (Fig. 4V, V').

Pygidium with one long median cirrus and two short, lateral lobes (Fig. 4X).

###### Remarks.

*Prionospioparapari* sp. n. is very similar to *P.fallax* in having a prostomium that is truncated on the anterior margin, a neuropodial postchaetal lamellae on chaetiger II being the same shape, and a high dorsal crest on chaetiger 7. However, *P.parapari* sp. n. can be distinguished from *P.fallax* as redescribed by Sigvaldadóttir and Mackie (1993), by the former having rounded (rather than rectangular) postchaetal neuropodial lamellae on chaetiger I, the first pair of branchiae longer than fourth (rather than of equal size), and with dense digitiform (rather than sparse lateral) pinnules arranged along the posterior face of the stems on branchiae 1 and 4, and cirriform (rather than subtriangular) second and third branchial pairs. In addition, the low dorsal crests in *P.parapari* sp. n. are present on chaetigers VIII–IX while in *P.fallax* they are absent; the neuropodium on chaetiger III in *P.parapari* sp. n. is oval with an enlarged dorsal edge whilst in *P.fallax* it is subtriangular and dorsally pointed; and the sabre chaetae in *P.parapari* sp. n. are heavily granulated and bilimbate whereas in *P.fallax* they are distally granulated and alimbate. The pygidium lacks pigmentation in *P.parapari*.

*Prionospioparapari* sp. n. is also similar to *P.komaeti* Hylleberg & Nateewathana, 1991, *P.depauperata* Imajima, 1990, and *P.oligopinnulata* Delgado-Blas, 2015, in that all three species have a prostomium that is truncated on the anterior margin, the same shaped neuropodial postchaetal lamellae on chaetiger II, and a high membranous dorsal crest on chaetiger VII that decreases in height on chaetigers VIII–IX. However, *P.parapari* sp. n. differs from the first two species in that it has rounded (rather than square or lanceolate) notopodial and neuropodial postchaetal lamellae on chaetiger I, and an oval neuropodium on chaetiger III with the dorsal edge enlarged (rather than one that is square or triangular). In addition, the branchiae of *P.parapari* sp. n. have long, naked, smooth distal tips, whereas those of *P.komaeti* and *P.depauperata* have pinnules extending to the distal end, and *P.parapari* lacks low dorsal crests on chaetigers X–XI/XIII. *Prionospioparapari* sp. n. also differs from *P.depauperata* in that it has sabre chaetae without a distal filament, the notopodial hooded hooks start on chaetigers XIX–XXXVIII rather than XLV–XLVII, and the pygidium has two long lateral lobes rather than two short lateral cirri. Furthermore, *P.parapari* sp. n. is similar to *P.oligopinnulata* in that both species show the same pygidial structure, but differs in having cirriform rather than triangular second and third branchial pairs. In addition, the low dorsal crests on chaetigers X–XIV are absent in *P.parapari* sp. n., the neuropodium on chaetiger III is oval with the dorsal edge enlarged (rather than subtriangular and ventrally pointed), and the sabre chaetae are bilimbate (rather than alimbate). *Prionospioparapari* sp. n. is similar to *P.rotunda* Delgado-Blas, 2015 in that both species have large, rounded neuropodial postchaetal lamellae on chaetiger I, cirriform second and third branchial pairs, the first branchial pair always longer than fourth pair, and the same pygidium structure. However, *P.parapari* sp. n. differs from *P.rotunda* in having a bottle-shaped prostomium that is truncated on the anterior margin (rather than a pyriform and rounded one). In addition, the low dorsal crests in *P.parapari* sp. n. are present on chaetigers VIII–IX whereas in *P.rotunda* they are absent, and the neuropodium on chaetiger III is oval with an enlarged dorsal edge (rather than rounded). The differences between this new species and the other species examined are provided in the key.

###### Etymology.

The species is named in honor of Dr Julio Parapar, in recognition of his major contribution to the study of polychaetes from Spanish coasts.

###### Type locality.

Ría de Ferrol and the mouth of Piedras River, Huelva, Spain.

###### Distribution.

To date, this species has been recorded only on the Spanish Atlantic coast (Ria de Ferrol and the mouth of Piedras River, Huelva).

#### Key to *Prionospio* species with four branchial pairs from the Iberian coastline

**Table:** 

1	First 3 pairs of branchiae apinnate and pair 4 with pinnules; or first pair of branchiae with pinnules and pairs 2–4 apinnate	**2**
–	First and fourth pair of branchiae pinnate and pairs 2–3 apinnate	**4**
2	First 3 pairs of branchiae apinnate and pair 4 with pinnules; dorsal crest on chaetiger VII; notopodial prechaetal lamellae very large on anterior chaetigers, basally fused with notopodial postchaetal lamellae; interparapodial pouches absent; all hooded hooks bidentate	*** P. caspersi ***
–	First pair of branchiae with pinnules and pairs 2–4 apinnate	**3**
3	Second and third branchial pairs slightly expanded distally, with short, sharp tips; a low crest on chaetiger V; sabre chaetae sheathed	*** P. ehlersi ***
–	Second and third branchial pairs triangular and thick, a dorsal cord-shape on chaetiger V; sabre chaetae alimbate	** P. cf. ehlersi **
4	Ventral crests present on chaetigers XI/XII–XV/XIX; high dorsal crests on chaetigers X–XI, and low dorsal crests on chaetigers III–IV, XII–XXII/XXXIV; notopodial prechaetal lamellae very large on anterior chaetigers, basally fused with notopodial postchaetal lamellae	***P.cristaventralis* sp. n.**
–	Ventral crests absent; high dorsal crest on chaetiger VII, and low dorsal crests on chaetigers VIII–IX or absent; notopodial prechaetal lamellae moderate or low on anterior chaetigers, not basally fused with notopodial postchaetal lamellae	**5**
5	Prostomium square; caruncle long; peristomium medium-length; second and third branchial pairs cirriform; dorsal crests on chaetigers VIII–IX; neuropodial postchaetal lamellae oval, ventrally directed on chaetiger II, and oval, dorsally directed on chaetiger III	***P.parapari* sp. n.**
–	Prostomium bottle-shaped; caruncle short; peristomium short; second and third branchial pairs subtriangular; dorsal crests absent; neuropodial postchaetal lamellae subtriangular, ventrally pointed on chaetiger II, and subtriangular, dorsally pointed on chaetiger III	*** P. fallax ***

## Supplementary Material

XML Treatment for Prionospio caspersi

XML Treatment for Prionospio cf. ehlersi

XML Treatment for Prionospio fallax

XML Treatment for Prionospio cristaventralis

XML Treatment for Prionospio parapari
